# The Consumers' League

**Published:** 1890-12-13

**Authors:** 


### THE CONSUMERS' LEAGUE.

By a Correspondent.

Those of our readers who have followed with any sympathy the course of our series of articles upon Working Women must have many a time felt their hearts heavy with the sense of the hardships and miseries of so many of their fellow-creatures; must have cried out that such a state of things should not be tolerated for an hour in this Christian land of ours: if only they could see how to put a stop to it. Well, we have already tried to show that efforts have been made, and are being made, to better, to some extent, at any rate, the condition of our working women. What we wish to do here, however, is to call special attention to a new departure that is about to be made—to the formation, namely, of what has been long thought of and talked over by friends of the working-classes —a Consumers' League. A meeting to arrange for this was held, as our readers probably know, at the Westminster Palace Hotel, on Wednesday, November 19th, Canon Scott Holland taking the chair; a working committee was then formed, and in a short time, no doubt, the work will be fairly entered upon.

First of all, let us understand why such a League is needed. For this reason—that the various investigations which have been entered upon of late by Special Commissions and otherwise, have shown us that it is we, the consumers, who are in reality responsible for much of the distress which has been revealed to our startled gaze. We have not really known this until lately. We believed in all sincerity that it was the middleman or the shopman who was in fault, that we were blameless, and he a monster of iniquity. Now, to our alarm and disgust, we find the burden of responsibility shifting from one to another, one to another, until it finally stops and rests upon our shoulders—upon us, the consumers. How can this thing be? we cry in shame, and in strong rebellion against our fate. And then we find that it is we who oppress the shopman, rather than—or perhaps we should say, as well as—he who oppresses the work-people; that we bring a terrible pressure to bear upon him which finally passes from him to the unfortunate producers of the articles we consume, and grinds them into the very dust.

Most unwittingly and unwillingly indeed have we done this, none the less we have done it—aye, and are doing it now. As Canon Scott Holland feelingly put it:—"It is bad enough to find that without our knowledge a long course of cruelty has been practised; to find that to our account this cruelty must be laid is simply intolerable. 'Thou art the man' has been said to us, as it was once said to David; and like David, we wince under it."

This does not mean that it is the rich alone—no, nor even the upper and middle classes alone, who are to blame. A member of the London Trades Council admitted at the meeting that working-men also were responsible, and were beginning to see that they were responsible in this matter; and it is, in fact, the whole body of consumers, rich and poor, who bring this pressure to bear upon the producers of the articles which they consume. This is no more than natural, when one comes to think of it. Given the fierce competition for work, which, as every one knows, prevails in this over-populated country of ours, and the intense eagerness to pick up cheap things which is so marked a feature of present day transactions, and it becomes almost self-evident that unless the consumer himself makes a stand, no one else can.

Unless the consumer himself makes a stand! Well, that is exactly what the Consumers' League proposes to do—to make a stand against the unthinking impulse which urges us to do the very best we can for ourselves, regardless of consequences; to "release the shopkeeper (we quote Canon Scott Holland again) from the tyranny of our ignorance," to substitute, and to urge others to substitute, more humane and Christian system of trade for that which has now been found to result in so much misery.

We believe that there are large numbers of consumers who, with the worthy Canon, are aghast at finding where the responsibility lies, and who honestly and eagerly long to know that if they force their way down the long line of persons engaged in producing their articles for consumption, they will find at the end a man or woman who will smile and shake hands with them over the bargain—ah, the picture brings tears to one's eyes, such a terrible contrast is there between it and the sad reality! But we believe—nay, we must say we are sure—that there are also many people who trouble themselves about nothing but their own profit, and do not stand for the sufferings of others, provided they are not brought into immediate contact with those sufferings; who declare that each man must do the best he can for himself, wilfully blinding their eyes to the fact that many poor workers are powerless to secure for themselves anything that can be called even "good," let alone "best," and that they are, in fact, deliberately living upon the flesh and blood of their fellow-creatures. If the League can rouse such people, and make them realise, without hope of escape, what it is that they are doing, it will not have been started in vain.

But the primary object of the League is, no doubt, to bind together all such consumers as are already honestly anxious to have none but fair dealings with those concerned in production; to make careful and judicious inquiries into the state of certain trades, and into the rate of wages and the conditions of labour of the employés in various firms; and then to make known to its members, and through them to the public in general, the names of such employers as are proved to deserve the technical appellation of "fair." In no sense is the League intended to work against shopkeepers and employers; it rather aims at helping them, by relieving them from the pressure which has ignorantly been brought to bear upon them by the very purchasers themselves; and it is hoped that they will look upon the League as a friend, not an enemy. One large employer of labour was present at the meeting, and supported one of the resolutions which were passed at it; and we do not see why good employers generally should not give their hearty support to the movement.

Much, of course, depends upon the tact and wisdom shown by the working committee, but much also depends—and for this reason we are anxious to lose no time in bringing the League before the notice of our readers—on the amount of support given to it by the public. As we pointed out at the meeting, shopkeepers are and must be the servants of the public, for they live by the pleasure of the public. If they find that that public is really anxious to support fair dealing depend upon it, those who can show that they do deal fairly will hasten to prove it to the League, while those who cannot will find it to their interest to introduce a different method into their business.

And speaking of interest, there is no reason, so far as we can see, why the changes which the League hopes to introduce should be for the interest of producers and retailers only. Cheap labour is bad labour; and we fully believe that in many cases the craze for cheap things which has seized upon the public only appears to save the consumers' pockets, while at the same time inflicting untold hardships upon the producer.

Doubtless the League has difficulties in its path. It cannot give the precise and detailed information which some people will probably clamour for—to do this and to show proper consideration to the employers would be impossible; neither can it always assure us that good wages are paid even by the firms which it recommends—in some trades, alas! it is to be feared that there are no really "good" wages; still, it can at least show us what houses deal fairly, as things go, by their employés, and this of itself is a great thing. It will pursue its labours carefully and steadily, publishing the results from time to time, and meanwhile, we trust that the public's support will be heartily given to it. Whether it fouls or whether it succeeds, we cannot doubt that it is a step in the right direction.

